# PReS13-SPK-1588: Recurrent fevers

**DOI:** 10.1186/1546-0096-11-S2-I20

**Published:** 2013-12-05

**Authors:** J Frenkel

**Affiliations:** 1 Paediatrics, University Medical Center Utrecht, Utrecht, Netherlands

## 

Fevers are common in childhood, usually due to infections.

Some children however experience recurrent episodes of seemingly unprovoked fevers. These so-called periodic fever syndromes are rare diseases. Clinically they are characterized by generalized inflammation and different combinations of localized tissue inflammation. Skin and joints are often affected in these patients and the long-standing inflammation can lead to irreversible organ damage due to tissue deposition of inflammatory amyloid proteins. Especially the kidney is vulnerable tot his so-called AA-amyloidosis. The spontaneous sterile inflammation in the absence of autoantibodies places the periodic fever syndromes in the same category as systemic juvenile idiopathic arthritis: the group of autoinflammatory diseases. Often these are genetically determined.

Over the past 15 years genetic defects have been identified underlying more than twenty such genetic autoinflammatory diseases. These are disorders like Familial Mediterranean Fever, caused by mutations in the *MEFV *gene, TNF-receptor associated periodic syndrome (TRAPS), caused by mutations in the *TNFRSF1A *gene and the Chronic Infantile Neurological Cutaneous Articular (CINCA) syndrome, caused by mutations in the *NLRP3 *gene.

Identification of the responsible genes has led to understanding of the pathophysiology and hence to effective targeted therapy. Interleukin-1 has proved to be the central mediator in many of these disorders. This finding in the congenital autoinflammatory diseases has led to novel therapies in more common disorders like interleukin-1 blockade in systemic Juvenile Idiopathic Arthritis. The rarity of the periodic fever syndromes hampers evidence based therapy. International collaboration in the EUROFEVER network has enabled us to better define the clinical picture of these disorders and to select targets for therapeutic research. However, many children currently defy genetic diagnosis. Next generation genetic sequencing efforts will hopefully identify the cause of inflammation in this group of patients.

The functional consequences of autoinflammatory diseases are primarily related to the unpredictable fever episodes. However irreversible sequelae, such as hypertrophic arthropathy, chronic renal failure, impaired vision or hearing do occur. Since these are largely preventable by adequate control of inflammation, effective therapy is essential.

## Disclosure of interest

None declared.

